# Population health and wellbeing: Identifying priority areas for Victorian children

**DOI:** 10.1186/1743-8462-2-16

**Published:** 2005-07-20

**Authors:** Elise Davis, Elizabeth Waters, Melissa Wake, Sharon Goldfeld, Joanne Williams, Ozlem Mehmet-Radji, Frank Oberklaid

**Affiliations:** 1 School of Health and Social Development, Faculty of Health and Behavioural Sciences, Deakin University, 221 Burwood Hwy, Burwood, Victoria, 3125, Australia; 2 Centre for Community Child Health, Royal Children's Hospital, Department of Paediatrics, University of Melbourne, Murdoch Children's Research Institute, Flemington Rd, Parkville, 3052, Australia

## Abstract

**Background:**

Population health information, collected using soundly-designed methodologies, is essential to inform policy, research, and intervention programs. This study aimed to derive policy-oriented recommendations for the content of a health and wellbeing population survey of children 0–12 years living in Victoria, Australia.

**Results:**

Qualitative interviews were conducted with 54 academic and policy stakeholders, selected to encompass a wide breadth of expertise in areas of public health and inter-sectoral organisations relevant to child health outcomes, including universities, government and non-government agencies across Victoria. These stakeholders were asked to provide advice on strategic priorities for child health information (data) using a structured interview technique. Their comments were summarised and the major themes were extracted. The priority areas of health and wellbeing recommended for regular collection include obesity and its determinants, pregnancy and breastfeeding, oral health, injury, social and emotional health and wellbeing, family environment, community, health service utilisation, illness, and socioeconomic position. Population policy questions for each area were identified.

**Conclusion:**

In contrast to previous population survey programs nationally and internationally, this study sought to extract contemporary policy-oriented domains for inclusion in a strategic program of child health data collection, using a stakeholder consultation process to identify key domains and policy information needs. The outcomes are a rich and relevant set of recommendations which will now be taken forward into a regular statewide child health survey program.

## Background

Epidemiological child health data are an essential driver for policy, advocacy, service design, health promotion and prevention programs. Such data can make population health strengths, deficits and inequalities explicit, provide evidence of the influence that the social and political contexts have on health [1] and provide evidence for improvement or worsening in health parameters over time. Data from population surveys may influence both the direction and content of policies and programs for government and non-government organisations, in areas such as education, health, transport, justice and the environment. In designing a population health survey for children, there is a potentially exhaustive list of areas of health that could be examined. It is important that a) the areas that are included reflect the changing mortality and morbidity patterns in children and the changing environments to which children are exposed and b) the results have the potential to inform policy and programs and are user-friendly. This paper will demonstrate a process of selecting areas of child health that meet these criteria, using preliminary work towards the development of a new survey on Victorian children as an example.

A recent review of national and international epidemiological studies of children's health and wellbeing demonstrated that these studies included several different areas of child health such as chronic diseases, physical activity, oral health, development, services, and neighbourhood and family interactions [2]. In developing a new population survey for children, it is possible to simply apply an existing survey to a new population. However, given that population surveys are resource-intensive and time consuming for families, there is an ethical obligation and financial benefit for survey developers to collect only information relevant to the specific population and the rationale for that survey. Unfortunately, there does not appear to be any standard procedure for identifying the relevant areas of health for a population. A recent review of the major national and international population surveys of child health and wellbeing has demonstrated that researchers often fail to report how they identified the relevant areas of child health [2].

In this paper, we propose that the first step in identifying the relevant areas of child health is to consider a comprehensive intersectoral approach to child health and wellbeing, and its determinants. Theoretical models of health and development, such as the social health model [3,4], ecological model [5,6], Lynch's model [7], the National Child Health Performance Framework [8,9] and the lifecourse perspective [10] are useful to understand the scope of children's health. For example, the ecological model proposes that children need to be considered within their family, school, neighbourhood and the larger social, structural, economic, political and cultural environment [5,7]. The National Child Health Performance Framework, developed by the National Health Performance Committee, contains a set of indicators to measure trends in health status, determinants of heath and the use and delivery of service [8,9]. Although these models are important in understanding the spectrum of child health, it is clearly not possible, and may not even be necessary, to include all of these indicators of child health in a population survey. The next step in developing a population survey is to prioritise the areas of child health relevant to the funders and users of population data.

It is possible to apply some criteria to reduce the potential list of areas. Only one epidemiological study of children's health has developed and reported criteria to prioritise areas of child health. The New South Wales Child Health Survey [11] selected their areas of child health using the following criteria: 1) it is a priority for child health as documented in a state or national child health policy document; 2) it meets the information needs of the NSW Department of Health and Area Health Services in relation to child health; 3) the information is not readily available from other sources; 4) the estimated sample size is large enough to provide data that can be used to generalise responses to the NSW population of children; 5) the areas are not highly sensitive to respondents and likely to cause failure to complete the survey.

The Strategic Plan Health Gain for Children and Youth in Central Sydney prioritised issues for children and youth by analysing information on prevalence, severity of a condition, community concern for the issue and efficacy of available interventions [12]. Other important criteria have been developed for use in adult population health surveys. According to the NSW Strategy for Population Health Surveillance, each area of health is considered in terms of its burden (ie. incidence, prevalence, mortality, years of potential life lost, hospitalisation rate), preventability, communicability, public interest, and legislative requirements [13].

In this paper we argue that the criterion through which all others need to be filtered is that the data have the potential to inform policy and programs. It is essential that survey developers consider dissemination and uptake of results, ensuring there is utility for researchers and policymakers alike. To address this criterion, it is recommended that survey developers consult with stakeholders and potential users of the data, and then apply the remaining criteria. Stakeholders can provide insights on policy and program decision making, the use of data in that process and recommendations for the best data. This paper aims to demonstrate a process by which survey developers can consult with stakeholders to determine the relevant areas of child health for a population survey of child health and wellbeing.

## Method

### Participants

Fifty four key stakeholders participated in this study. The sample was selected to represent the areas of health and development in the National Child Health Performance Framework [8,9]. The National Child Health Performance Framework consists of three broad groups of indicators health status, risk and protective factors, and services and interventions. Health status has four subgroups: health and wellbeing, growth and development, mortality, morbidity and disability, and safety and security. The risk and protective factors group has three subgroups: social, cultural and environmental factors; biological and behavioural factors and health knowledge. The services and interventions group includes health services, health programs, health promotion and intervention, intersectoral services and community services.

To identify the indicators of health status, we consulted with stakeholders with expertise in children's physical, social and emotional wellbeing, development, disability, mental health problems, illness, oral health problems, nutrition related problems, child abuse and parental health and wellbeing. To identify the risk and protective factors, we consulted with stakeholders with an understanding of the impact of the physical, family, economic, social and school environment. We also consulted with experts in the area of child diet, activity and overweight and obesity. To identify services and interventions, we consulted with stakeholders with knowledge about health service utilisation, maternal and child health programs, community services and health promotion programs.

Stakeholders were selected to encompass a variety of organisations, including university and government departments within Victoria. The stakeholders were identified by the authors and though literature reviews. A snowball technique was also used where initial respondents were asked to suggest others whom they know are in the target group and who could be invited to take part, and so on.

### Materials

The stakeholders participated in one-on-one interviews. The interviews were semi-structured and the questions were adapted from those included in a quasi-delphi study for the Victorian Adolescent Health and Wellbeing Survey [14,15]. The stakeholders provided advice on the area of child health that they have expertise in, what they thought were the most important areas of health and discussed how they would use the results (Refer to Table 1).

**Table 1:** Interview questions for stakeholders

1) Which key areas of child health are you interested in?
2) Thinking about current policy and programs within the key area, which specific aspects of children's health and wellbeing would you measure in a statewide survey?
3) Would your organisation use the results of a statewide survey of children's health than measured these aspects? If so, how? What results are needed?
4) In what format would you want to receive the results so that they were meaningful for you?

### Procedure

The interviews with the stakeholders generally lasted between 15–45 minutes. Interviewers recorded the major points of the interview, and produced a summary of each interview. The summaries were then sent to the interviewees to correct information and/or add further information. Once corrected, the responses for each question were entered into an Excel database and the data was coded by two researchers using open coding. This is the process of identifying persistent words, phrases, themes or concepts within the data so that the underlying patterns can be identified and analysed [16]. A coding framework was developed and two researchers coded each of the summaries using focused coding (EW, ED). Agreement on key themes was achieved by discussion.

## Results

Fifty-four stakeholders participated in this study. Stakeholders were asked which aspects of child health they were interested in. As demonstrated in Table 2, their areas of interest could be mapped to the National Health Performance Framework. The total number of areas exceeds 54, because several stakeholders indicated more than one area of interest/expertise.

**Table 2:** Stakeholders expertise

**Indicators of NHPF**	**Subgroups of NHPF**	**Stakeholders Areas of Expertise (numbers of stakeholders)**
Health Status and Outcomes	Life expectancy and wellbeing	Child physical (4) Child social and emotional wellbeing (9)
	Mortality, morbidity and disability	Child disability (3) Child mental health problems (5) Childhood injury (2) Child chronic illnesses (4)
Risk and Protective factors	Environmental factors	Physical environment (2) Community environment (4) Exposure to tobacco smoke (4)
	Socioeconomic factors	Economic environment (3) Child education (5) Parental employment (1)
	Community capacity	Family environment (9) Social environment (7) Parental health (1)
	Health behaviours	Health behaviours – All (2) Child physical activity (4) Child diet and nutrition (3) Child oral health behaviours (2) Sun protection (1) Vaccinations (1) Injury prevention (2)
	Person-related factors	Birth defects (1) Health behaviours during pregnancy (ie smoking, alcohol, folate) (2)
Services and Interventions		Health service utilization (3) Maternal and Child Health Programs (1) Community services (1) Health promotion programs (1)
Socio-demographic factors		Socioeconomic position (2) Socioeconomic inequalities (2) Family structure (1)

Population groups		Socioeconomically disadvantaged groups (4) Rural and remote area residents (1) Overseas born (1) Indigenous Australians (1)

NHPF – National Health Performance Framework

Stakeholders were also asked what aspects of child health they would include in a population survey of Victorian children's health and wellbeing. As several different areas of health were identified, their responses were grouped according to major themes. These include obesity and determinants, social and emotional health and wellbeing, family environment, health service utilisation, illness, community, oral health, injury, pregnancy and breastfeeding and socioeconomic position. Table 3 demonstrates the overarching themes, the areas that represent the themes, and the specific data that are required by the stakeholders.

**Table 3:** Priority Areas of Child Health Identified by Stakeholders

**Themes**	**Areas of Child Health**	**Specific data required by stakeholders**
Obesity and determinants	Physical activity Nutrition Obesity	1) Need epidemiological data on childhood obesity, physical activity, sedentary behaviours and nutritional intake in Victoria. 2) Need data on mediating and psychosocial variables.
Social and emotional health and wellbeing	Social and emotional wellbeing Behavioural problems Mental health	1) Need data on the prevalence and distribution of mental health problems. 2) Need data on the adequacy of mental health services and barriers to seeking help.
Family Environment	Family environment Parenting style Reading Exposure to smoking	1) Families have undergone substantial changes, and we need data on how different family environments impact on children's health.
Health service utilisation	Health service utilisation	1) We need data to ensure that our services are meeting the needs of the community, and ensure that people are satisfied with them.
Childhood illness	Chronic illness Disability Development	1) Need data on the prevalence of chronic illness and disability. \par
Community	Neighbourhood/Community	1) The community environment impacts on children's health; to get a complete picture of children's health, need to examine the community environment.
Oral health	Oral health	1) There are no population data on the oral health status of children, across this proposed age group.
Injury	Injury	1) Need data on the prevalence of injuries and how they are treated. 2) Need data on whether families are reducing the risk of injuries by protecting their home.
Pregnancy and breastfeeding	Breastfeeding Smoking in pregnancy	1) Need prevalence data on smoking, alcohol and folate intake during pregnancy.
Socioeconomic position	Health inequalities	1) A statewide survey of child health should include the child's socioeconomic position to examine distributional effects of health and program effectiveness.

After gaining insight into the potential for each area of child health to aid policy and program decisions, the remaining criteria can now be applied. Based on the criteria developed for the NSW Child Health Survey and the criteria developed for the NSW Strategy for Population Health Surveillance, it is recommended that:

1) The information is not being collected elsewhere (ie. databases, school records etc).

2) The question can be answered using a population survey.

3) The domain impacts on children's mortality or morbidity.

4) The area of child health can be measured in a population survey (ie depending on data collection method and length).

5) The data are user-friendly and the results have the potential to inform policy and programs

Using these criteria, breastfeeding, development and parenting style were excluded. Breastfeeding is already being measured by maternal and child health centres and the Australian Bureau of Statistics. Although child development is important, assessments of children's developmental status are extremely resource intensive and therefore unable to be employed in a population data collection. Parenting style was assessed by the authors to be less useful for policy and program development. The remainder of the areas met the above criteria, and were therefore included.

The stakeholders indicated that the results from a child population health survey could be used in the pursuit of evidence-based policies, practice and programs, for service planning, for advocacy, to develop networks across community, to support the generation of appropriate local responses, to develop interventions, to use in submissions for funding, and to use in publications. Some stakeholders indicated the need to localise data and to make it more powerful in action terms; other stakeholders suggested that rural/urban comparisons would be important. Stakeholders suggested that the results should be available from both a representative sample and also from key minority groups such as Indigenous children. The results should also contain some comparable measures to other work done elsewhere.

## Discussion

This study demonstrated the process by which areas of child health can be identified and prioritised for a population study of health and wellbeing. Conducting qualitative interviews with stakeholders is a useful and efficient method to identify current issues in a specific area, and to provide exposure to significant research papers and unpublished research. The areas of child health that were identified in this study are not only useful in developing a population survey of child health and wellbeing for Victorian children, they are also useful for researchers and practitioners in the field of child health, in terms of guiding research, policy and program development.

The main themes of child health tended to reflect the changing patterns of morbidity, where there is increasing interest in the rising prevalence rates of obesity, mental health problems, and oral health problems. The emphasis on health service utilisation, disability and chronic illness is reflective of the costs that such children impose on the health care system. The emphasis on family health, exposure to tobacco smoke, community and socioeconomic position is indicative of the more recent emphasis placed on the wider community environment and influences, and recognition that children's environments have changed profoundly. In terms of the specific data that the stakeholders recommended for each area of health, there was a clear need for prevalence data and also for establishing and modelling the determinants of child health.

The areas of child health that emerged from the interviews are consistent with the stakeholders' areas of expertise. Although it seems likely that the exact sample of stakeholders will always influence the areas of child health that are identified, the selection of these stakeholders was based on the National Child Health Performance Framework, an acceptable indicator framework.

In terms of the process by which researchers determine the priority areas of child health, we recommend that survey developers utilise a model of health and development, such as the National Child Health Performance Framework, to identify the possible areas of child health. To prioritise areas, it is recommended that survey developers consult with relevant stakeholders to ensure that the data are user-friendly and the results have the potential to inform policy and programs. The selection of the stakeholders' areas of expertise should be consistent with a selected theory or framework of health. The areas of child health identified by the stakeholders can be further prioritised using the proposed criteria, which are based on the NSW Strategy for Population Health Surveillance.

## Limitations

This study has limitations in its sampling methodology. The stakeholders were identified by the authors and through the use of snowballing. This methodology does have potential for selection bias and thus may limit generalisability of results. Given that the stakeholders were selected to ensure that there were representatives from all areas of health, selective sampling was necessary.

A further issue for discussion is the inclusion of children in such a study. Increasingly there is recognition that children and parents need to be included in program planning and policy development. Given the format of the questions and the aim of this study, children's and parent's perspectives were not obtained. It is recommended that when the questionnaire is established and parents and children can understand what is being measured, they should be consulted about the areas of health that are included in a population survey. This process is currently being undertaken with a diverse group of parents and children.

## Conclusion

Population child health data is important for informing policies, programs and services in a range of sectors. However, the process by which researchers determine the priority areas of child health remains largely un-defined. The phases of this study included a rigorous research process, including qualitative interviews with stakeholders in the area of child health.

## Competing interests

The author(s) declare that they have no competing interests.

## Authors' contributions

All authors made substantial contribution to the conception, design, analysis and interpretation of data. ED and JW conducted the data collection. ED and EW analysed the data for themes. All authors read and approved the final manuscript.
